# The Perceived Impact of COVID-19 among Treatment-Seeking Smokers: A Mixed Methods Approach

**DOI:** 10.3390/ijerph18020505

**Published:** 2021-01-09

**Authors:** Zoe Rosoff-Verbit, Erin Logue-Chamberlain, Jessica Fishman, Janet Audrain-McGovern, Larry Hawk, Martin Mahoney, Alexa Mazur, Rebecca Ashare

**Affiliations:** 1 Department of Psychiatry, Perelman School of Medicine at the University of Pennsylvania, Philadelphia, PA 19104, USA; rzoe@pennmedicine.upenn.edu (Z.R.-V.); loguerin@pennmedicine.upenn.edu (E.L.-C.); fishman1@upenn.edu (J.F.); audrain@pennmedicine.upenn.edu (J.A.-M.); maza@pennmedicine.upenn.edu (A.M.); 2 Annenberg School for Communication, University of Pennsylvania, Philadelphia, PA 19104, USA; 3 Department of Psychology, State University of New York at Buffalo, Buffalo, NY 14260, USA; lhawk@buffalo.edu; 4 Department of Internal Medicine, Roswell Park Cancer Institute, Buffalo, NY 14263, USA; Martin.Mahoney@RoswellPark.org

**Keywords:** smoking cessation, tobacco use, theory of planned behavior, COVID-19, coronavirus

## Abstract

The consequences of the COVID-19 pandemic on behavioral health, including tobacco use, are not fully known. The current study sought to measure the perceived impact of COVID-19 and the resulting stay-at-home orders in Philadelphia, Pennsylvania and Buffalo, New York on smokers enrolled in four smoking cessation trials between March 2020 and July 2020. The survey collected quantitative data regarding life changes due to COVID-19, health/exposure status, and the impact on their cessation attempt (e.g., motivation to quit, change in triggers). The questionnaire collected qualitative data to better understand how such changes could explain changes in smoking behavior. Of the 42 participants surveyed, approximately half indicated that COVID-19 changed their motivation and ability to quit or remain quit. Among those who reported that it was easier to quit following the stay-at-home orders (*n* = 24), most attributed this to concerns regarding the severity of COVID-19 among smokers. Among those who reported more difficulty quitting (*n* = 15), most attributed this to their increased stress due to the pandemic and the inability to access activities, places, or people that could help them manage triggers. Given public health warnings of continued surges in COVID-19, these data provide insight into who may benefit from further smoking cessation support should existing restrictions or new stay-at-home orders be enacted.

## 1. Introduction

The rapid spread of the SARS-CoV2 virus, which causes the illness COVID-19, resulted in the World Health Organization (WHO) declaring it a pandemic on 11 March 2020. To date, the virus has claimed over 1 million lives globally with more than 38 million confirmed cases [1]. In the United States, the country with the highest number of confirmed cases and deaths, more than 350 thousand people have died and more than 20 million have been diagnosed with COVID-19 [1,2]. In addition to the overwhelming toll on human life, COVID-19 has caused a profound disruption in the global economy, has dramatically altered the way we work, play, and interact with family and friends, and has contributed to an overall increase in levels of distress.

To reduce the spread of the virus, health organizations and government officials have recommended reducing physical contact, such as keeping at least six feet of space between oneself and others, commonly referred to as “social distancing”, wearing a mask or other facial covering when in public, and more frequent hand washing [3]. Nearly all states in the United States issued some type of “stay-at-home” or “shelter in place” order to promote social distancing [4]. Although the specific rules vary by state, and in some cases, by county within specific states, in general, these orders required or recommended that residents stay home except for essential travel, placed restrictions on large gatherings, and either temporarily closed many businesses or required them to operate at reduced capacity. Philadelphia (Philadelphia county, Pennsylvania) and Buffalo (Erie county, New York) both had similar stay-at-home orders issued around the same time. In both cities, the stay-at-home order asked residents to remain home unless purchasing essential goods or seeking medical attention, and permitted only essential businesses to remain open, while allowing certain outdoor activities as long as social distancing could be maintained [5,6].

In addition to the staggering effect the pandemic has had on physical health, the measures taken to reduce the spread of the pandemic (e.g., stay-at-home orders) have exacted a burden on behavioral and mental health that may have lasting effects. For example, anxiety about contracting COVID-19, worry about family and friends getting sick, and grief from losing a loved one to COVID-19 have all contributed to widespread reports of poor mental health during the pandemic [7,8]. Moreover, social distancing requirements, closed businesses creating job loss and financial strain, and restrictions on where people can go and when, have all compounded the stress surrounding the pandemic [9]. Public health officials have warned that the social distancing guidelines and stress surrounding the pandemic may contribute to poor mental health outcomes, including substance use [10].

Unfortunately, those with substance use disorder, including tobacco, alcohol and other drugs, may be more susceptible to COVID-19 exposure and at higher risk of severe COVID-19 illness [10]. Potential increases in tobacco use are concerning in light of evidence that smoking is associated with increased severity of COVID-19 [11,12]. Early surveys evaluating changes in tobacco use suggested a mixed pattern of responses, with some reporting an increase in smoking cessation and others reporting an increase in use [13]. A study conducted in Italy in April 2020 found that cigarette consumption decreased and thoughts about quitting were most common among tobacco smokers (vs. those who use other tobacco products) [14]. However, few studies have evaluated the impact of COVID-19 on smoking behavior while smokers are actively engaged in a quit attempt. The current study surveyed smokers enrolled in four different smoking cessation studies at two universities, one in Philadelphia, Pennsylvania and one in Buffalo, New York, to assess the general perception of COVID-19 risk and the impact of stay-at-home orders. The main goal of the study was to better understand how perceptions of COVID-19 might impact their smoking cessation attempt.

This study was informed by the Theory of Planned Behavior [15], a model that has identified the determinants of a wide variety of behaviors including smoking and other substance use [16,17]. According to this model, behavior is most proximally influenced by levels of motivation (also called behavioral intention). Thus, we investigated if participants experienced a change in their quit-motivation levels. Specifically, we studied whether, during the stay-at-home order, participants experienced a change in their perception of control over their ability to quit, cope with stress, access to cigarettes and social support. We examined what factors participants reported as making their quit attempt easier or more difficult during the stay-at-home order. Lastly, we examined the beliefs underlying one’s attitudes towards a behavior of interest (i.e., behavioral beliefs), which represent perceived advantages or disadvantages to quitting or smoking during these circumstances.

As public health officials warned of a potential surge in COVID-19 cases during the fall and winter months, which resulted in new stay-at-home orders, these data may provide important insight into the factors that may represent risk and protective factors for tobacco use and may guide the development of cessation interventions tailored to times of crises.

## 2. Materials and Methods

### 2.1. Participants

Individuals were asked to participate in a 15–20 min survey if they were currently enrolled in one of four smoking cessation trials at the time the stay-at-home order went into effect: three at the University of Pennsylvania (UPenn) (NCT03712098, NCT03169101, NCT03254433) and one at the University at Buffalo (UB) (NCT03262662). Participants in all studies received smoking cessation counseling, combined with one of the following: transdermal nicotine patch (2 studies), varenicline (1 study) or an investigational study medication or placebo (1 study). Counseling manuals for all four studies followed public health service clinical guidelines [18]. All sessions were one-on-one and delivered by trained counselors who were supervised by a clinical psychologist. Following the stay-at-home orders, all sessions were conducted remotely.

### 2.2. Data Collection

All survey respondents had completed all, or part, of their study treatment during the time of their respective state’s stay-at-home order. The COVID-19 and Quitting Smoking survey was voluntary and the decision to participate did not influence their participation in the smoking cessation trial in which they were enrolled. Participants were contacted by phone and gave verbal consent to complete the questionnaire. Participants contacted three times without responding were deemed lost to follow up for the survey (they were not withdrawn from the clinical trial for failure to complete the survey). Demographics and smoking history were collected as part of each trial. All study protocols were approved by the respective institutional review board at UPenn (IRB protocol #s: 824860, 831835, and 825425) or UB (IRB protocol #: STUDY00000911).

Surveys were administered through the Research Electronic Data Capture (REDCap) tool [19,20]. Participants at the UPenn site completed the survey over the phone as a recorded interview with study staff. Participants at the UB site were given the survey link to complete independently. For both sites, the stay-at-home order was issued on 22 March 2020. Survey data were collected from May 2020 through July 2020.

The survey (available upon request) consisted of thirty-eight items, divided into three sections. The first twenty-three items of our survey measured current health/exposure status and life changes due to COVID-19 using the Coronavirus Health Impact Survey (CRISIS) Adult Self-Report Baseline Form V0.3 (items 20–29, 32–41 and 43–45, respectively), distributed by the National Institutes of Health (NIH) [21]. The next section of the survey asked participants how COVID-19 and their area’s stay-at-home order may have changed their smoking behavior and cessation attempt (e.g., Has the Coronavirus/COVID-19 crisis impacted your motivations for quitting smoking/staying quit?). The last section of the survey screened for depressive symptoms and generalized anxiety using the Patient Health Questionnaire-2 item (PHQ-2) and Generalized Anxiety Disorder-2 item (GAD-2) [22,23]. Mental health resources were offered to any participants who scored ≥3 on the PHQ-2 or GAD-2.

### 2.3. Data Analysis

Demographic and smoking characteristics were compared between sites (UPenn and UB) using chi-square tests for categorical variables and *t*-tests for continuous variables. Frequencies for all close-ended survey responses (e.g., Likert scale items) were evaluated using STATA version IC/64 [24]. In a supplementary analysis, correlations among variables from the CRISIS survey, PHQ-2, GAD-2 and number of days between the stay-at-home order and survey completion were conducted. Open-ended survey responses were transcribed by hand using the available audio-recordings and were checked for accuracy by another staff member. Two primary coders (ZRV, EKL) analyzed the open-ended responses from the surveys completed online and those that were transcribed using NVivo version 12 [25]. A third coder resolved any discrepancies in coding (JF). A thematic analysis was conducted after coding was completed.

The Theory of Planned Behavior [15] has shown that two psychological constructs help explain behavior change including smoking cessation: attitudes and self-efficacy [16,17,26]. These two constructs include the beliefs that underlie attitudes and self-efficacy towards performing a behavior. Therefore, qualitative data were coded for the beliefs that underlie attitudes and self-efficacy towards smoking and smoking cessation during the pandemic. The beliefs underlying attitudes (i.e., behavioral beliefs) were defined as the perceived advantages and disadvantages (i.e., positive and negative consequences) of smoking and of quitting smoking. These four categories of behavioral beliefs were initially included but due to substantial overlap in the codes, only advantages to smoking and advantages to quitting were kept. Self-efficacy beliefs were defined as those statements about circumstances that made it easier or more difficult to avoid smoking. For each of the codes described above, the results report on the beliefs most commonly reported (i.e., at least 10% of participants). Beliefs that were rarely reported are considered relatively idiosyncratic and are not discussed below [27].

## 3. Results

### 3.1. Participants

Overall, 42 subjects across both sites were surveyed. The survey response rates were 63% at UPenn (17 complete, 10 missed) and 50% at UB (25 complete, 25 missed) and were not significantly different (Χ^2^ = 1.2, *p* = 0.28). As shown in Table 1, there were site differences in race, self-reported income, marital status, employment status and smoking rate. The participants at the UPenn site were more likely to self-identify as Black/African-American, had lower self-reported income, were less likely to be married and employed, and smoked fewer cigarettes per day.

### 3.2. Mental Health during COVID-19 

Table 1 contains the average scores for the PHQ-2 and GAD-2 across both sites. A score ≥3 on the PHQ-2 and GAD-2 is considered clinically significant for depressive symptoms and generalized anxiety disorder, respectively. Overall, four participants (9.5%) had a score ≥3 on the PHQ-2 and seven participants (16.7%) had a score ≥3 on the GAD-2.

### 3.3. COVID-19 Exposure Status and Life Changes Due to COVID-19

At the time of survey completion, one participant (2.3%) reported having been suspected of having COVID-19 and 10 (23.8%) reported that a family member or friend had been diagnosed with COVID-19 (only two of which were household members). Across both sites, three participants (7.1%) reported that a family or friend died as a result of COVID-19, three (7.1%) reported that a family member or friend had been hospitalized due to COVID-19, nine (21.4%) reported a family member or friend lost their job, and six (14.3%) reported a family member or friend had to self-quarantine.

Table 2 provides a summary of life changes due to COVID-19. Overall, 50% of the sample reported being at least moderately worried about getting COVID-19 and 55% reported being at least moderately worried about a friend or family member getting COVID-19. About 40% and 45% of participants reported being at least moderately worried about their physical and mental health, respectively, and 50% reported experiencing at least moderate stress due to restrictions on leaving home due to COVID-19. All participants reported reading or talking about COVID-19, with more than half of the sample describing this as often or most of the time.

Although 45% (*n* = 19) of the sample reported having at least a little difficulty following social distancing recommendations, all but one person said that they had less contact with people outside their home and half the sample (*n* = 21) reported only leaving their home 1–2 days per week since the stay-at-home order was issued. Eleven people (26%) reported that their workplace closed, or they were not working since the stay-at-home order was issued. About 36% of participants indicated that the stay-at-home order had created at least moderate financial strain and 21% reported at least moderate concern about the stability of their living situation. Nine people (*n* = 7 at UPenn; site difference, Χ^2^ = 6.6, *p* = 0.01) indicated that they were concerned about food instability. With the exception of concerns about food instability, there were no significant differences across site for any of the variables reported above.

As shown in the Supplementary analysis, being worried about mental health because of COVID-19 was positively associated with being worried about either oneself or one’s family/friends getting COVID-19 (*r* = 0.40 and 0.36, *p* < 0.001 and *p* = 0.01, respectively), worry about physical health because of COVID-19 (*r* = 0.40, *p* < 0.001), and the frequency of reading/talking about COVID-19 (*r* = 0.37, *p* = 0.01). In addition, worry about mental health was positively correlated with actual PHQ-2 and GAD-2 scores (*r* = 0.31 and 0.44, *p* = 0.02 and *p* < 0.001, respectively). The number of days between the stay-at-home order and survey completion was not correlated with any other survey or demographic variable.

### 3.4. Smoking Status during COVID-19

As participants for this study were recruited from four different clinical trials, participants were at different points in their quit attempt at the time of survey completion. The average duration between the stay-at-home order and survey completion was 72 days (range: 43 to 120). All participants completed the survey after their target quit date (TQD) and most (*n* = 32, 76%) had their TQD prior to the stay-at-home order. Overall, 26 (62%) participants reported that they were not smoking at all prior to the stay-at-home order, but five participants indicated they had resumed smoking by the time they completed the survey. In total, at the time of survey completion, 30 participants (71%) reported that they were not smoking at all. Thus, 14 participants reported a change in their smoking behavior between the stay-at-home and the time of survey completion (five relapsed and nine quit).

### 3.5. Commonly Reported Self-Efficacy and Behavioral Beliefs about Smoking during COVID-19

The behavioral beliefs (i.e., the perceived negative and positive consequences, or advantages and disadvantages) most commonly reported about quitting and smoking during the pandemic are described in Section 3.8 and Section 3.9. As shown in Table 3, more than half the sample (54%) stated that the pandemic impacted their motivation to quit smoking. Many participants voiced that their motivation to quit smoking had increased, primarily due to health concerns regarding contracting COVID-19. Those who had decreased motivation to quit attributed this to boredom and difficulty staying active due to stay-at-home orders. Of the 20 participants who said the pandemic influenced their ability to quit smoking, 12 said it was more difficult, 3 said it was less difficult, and 5 said that some aspects have made their quit attempt more and less difficult at times. Participants reported that having less access to social support or strategies they were previously using to help them quit made it more difficult to quit/stay quit, whereas having less access to cigarettes or less social situations with smoking made it easier to quit/stay quit. We also coded strategies to manage triggers and cravings, which were categorized as strategies that a participant was using before the stay-at-home order was issued (pre-COVID strategies) and strategies that were developed since the stay-at-home order took effect (post-COVID strategies).

### 3.6. Self-Efficacy Beliefs: Factors That Made It More Difficult to Quit (n = 15)

#### 3.6.1. Not Having Access to Activities, Places, or People that Helped Manage Triggers (*n* = 2)

For example, this could include not being able to go to gym or visit friends and family who are supportive in them quitting. One participant put it this way: “I am not able to be active and go to the gym now, so that is a big trigger for me because staying active stopped me from smoking as much and staying busy stopped me from smoking, so that’s a trigger for me…” (ID#1, UPenn, female).

#### 3.6.2. More Opportunity to Smoke (*n* = 8)

Multiple participants attributed the pandemic and stay-at-home order to increased time at home and increased boredom, therefore creating more opportunity to smoke. One respondent indicated: “Boredom is one of the triggers that induces the urge to smoke. The stay-at-home order creates a lot more boredom.” (ID#36, UB, male).

#### 3.6.3. Stress (*n* = 10)

Another factor that many participants contributed as being a barrier toward quitting, or staying quit, was stress. Some people mentioned that the pandemic was responsible for new, or more stressful situations, while others attributed financial hardships to their overall stress. One participant who relapsed during the pandemic stated: “Stress got the best of me and I turned back to cigarettes to feel relaxed. Which I am so disappointed in myself about.” (ID#25, UB, female). Another participant who relapsed stated specific factors that contributed to stress: “More stress from not being able to live normal life. Working from home. Kids at home all day while I’m working. Easier to smoke, more opportunity” (ID#29, UB, female).

### 3.7. Self-Efficacy Beliefs: Factors That Made It Easier to Quit (n = 24) 

#### 3.7.1. Increased Motivation (*n* = 6)

Of those who reported a change in their motivation, most reported that the pandemic increased their motivation to quit/stay quit for health reasons (i.e., not contracting COVID-19, or understanding the dangers of smoking and contracting COVID-19). For example, one participant who quit smoking after the stay-at-home order stated: “I was more motivated to not smoke due to the coronavirus.” (ID#10, UPenn, male).

#### 3.7.2. More Time at Home with Family (*n* = 4)

Some participants mentioned that due to stay-at-home orders, they are spending most of their time with family who support their quit attempt. “The reason it’s been easier for me to not smoke is because I’m around my family more, and my family didn’t even know that I smoked… it’s just [like] easier because—like I said—I didn’t want to smoke in front of them.” (ID#8, UPenn, male). Another participant mentioned: “Stay-at-home orders had me basically stuck in the house 24/7 with my husband (who has been the biggest advocate and support in my quitting and is not a smoker)” (ID#34 UB, female).

#### 3.7.3. Less Access to Cigarettes (*n* = 6)

Participants who reported having less access to cigarettes voiced different reasons for this. Some attributed this to social distancing and stay-at-home orders not allowing them as much interaction with smokers. One individual described their situation as such: “I’m not in those situations where you can literally just walk up to someone with a cigarette and [you know] if you’re feeling the urge you can simply ask [you know] to buy one or if they have one.” (ID#9, UPenn, male).

Similarly, spending less time around other smokers or in social situations with people who smoke afforded participants less exposure to smoking and having cigarettes readily available to them. One participant who quit smoking attributed the stay-at-home order to their success: “Social distancing has been a boon for my quitting smoking.” (ID#34, UB, female). Other participants reported not going to the store to buy cigarettes, perceiving the purchase as non-essential and the threat of contracting COVID-19 being too high. Although cigarettes may still have been available throughout the pandemic, people had less access to them in the home by not purchasing them elsewhere. One participant who quit smoking after the stay-at-home order stated: “I did not want to go out and to get any cigarettes because I respect the stay in the house order. That helped motivate me to push myself to stop.” (ID#13, UPenn, male).

#### 3.7.4. Fewer Exposures to Triggers (*n* = 5)

Many expressed that triggers they experienced pre-pandemic were less relevant during the pandemic. For example, people reported driving less and many bars (especially early in the pandemic) were closed, providing fewer exposure to alcohol which is a common trigger. Having less exposure to situations that make someone want to smoke can facilitate their smoking abstinence. When asked if the pandemic and stay-at-home order had made quitting more or less difficult, one participant said: “Easier. Way easier. There’s nobody at the bus stop smoking.” (ID#10, UPenn, male).

### 3.8. Behavioral Beliefs about Advantages to Quitting (n = 7)

Many participants expressed that the likelihood of getting infected with COVID-19 and the severity associated with the disease, specifically in smokers, changed their attitudes around quitting. Some felt that it was advantageous to quit in order to prevent contracting the disease or experiencing severe COVID-19 symptoms. Participants expressed that the pandemic put their smoking behavior into perspective: “Well because of the virus and the information I am getting back with the COPD and with me having asthma is that I am easy to come into contact with the COVID virus and that motivated me to push myself to stop.” (ID#13, UPenn, male). Another person said: “Motivation to stay quit has increased immensely, as the virus is reported to be more deadly to smokers.” (ID#28, UB, male).

### 3.9. Behavioral Beliefs about Advantages to Smoking (n = 8)

A common message was that smoking was advantageous during the pandemic to help cope with stress. Smoking provided a way for individuals to feel more relaxed or less stressed which was also identified as a self-efficacy belief about quitting (Section 3.6.3). To illustrate, participants shared: “I turned back to cigarettes to feel relaxed.” (ID#25, UB, female). Another participant noted: “I just feel like I’ll be a little relaxed and a little less stressed [after smoking].” (ID#7, UPenn, female).

## 4. Discussion

The current study sought to assess the life changes, COVID-19 exposure status, health risk perception, and perceived impact on smoking behavior among participants currently enrolled in smoking cessation trials in two east coast cities in the United States. Overall, despite the fact that few participants in our study reported either having COVID-19 or having a family member or friend who had been diagnosed with COVID-19, most participants indicated that they were worried about either themselves or family/friends becoming infected. In addition, most participants reported that they were worried about their physical or mental health and had experienced stress due to COVID-19. With respect to smoking, more than half the sample indicated that the pandemic had changed their motivation to quit. Given that some participants reported an increase in motivation to quit and others reported a decrease, we utilized our qualitative data to understand why.

Both increases and decreases in smokers’ consumption have been documented during the pandemic. For example, of 582 exclusive cigarette smokers surveyed in Italy, more than two-thirds reported that their cigarette consumption had changed (either increased or decreased) [14]. Another study conducted in the United States found that 28% of tobacco users decreased use and 30% increased tobacco smoking [13]. The present study adds to this literature by documenting that over half of the current smokers report a change in their motivation to quit since the start of the pandemic. Some reported that the stay-at-home orders increased their smoking cessation motivation, while others reported a decrease.

Participants’ motivation to quit (or stay quit) during the pandemic and stay-at-home restrictions were largely related to their self-efficacy beliefs about what made quitting smoking more or less difficult. Consistent with the Theory of Planned Behavior’s notion that self-efficacy plays a large role in behavior change, our qualitative analysis revealed several salient self-efficacy beliefs about smoking and quitting smoking during the pandemic. For instance, some participants suggested that spending more time at home with family who supported their quit attempt, having less exposure to smoking triggers, and less access to cigarettes facilitated their quit attempt. In contrast, others indicated that pandemic-related stress, boredom, and less access to activities and places to help manage triggers all served as barriers to quitting.

Another factor identified by several participants that may have strengthened their motivation to quit is COVID-19 risk perceptions. Several participants indicated that reports of increased severity of COVID-19 illness among smokers may have increased their motivation to quit or stay quit. While the risk of COVID-19 illness from smoking is currently unclear, smoking is associated with the development of chronic health conditions, such as lung disease, cardiovascular disease and diabetes, which increase the risk of severe COVID-19 illness resulting in hospitalization and intensive medical care [28]. Moreover, there are substantial racial and ethnic disparities in the impact of COVID-19 and pandemic-related restrictions. For example, age-adjusted hospitalization rates for Hispanics, non-Hispanic Blacks, and non-Hispanic American Indian or Alaska Natives are about 4-fold greater compared to non-Hispanic Whites and non-White populations have demonstrated greater increases in excess deaths between January and October 2020 [29]. Moreover, infection rates among Blacks and Latinos are substantially higher than Whites [30]. Although social and structural determinants of health contribute to these disparities, our study did not explicitly measure what role these factors may have played in participants’ risk perceptions or quit attempt.

This study has a few limitations that warrant mention. All survey responses were self-reported and smoking abstinence was not biochemically verified. Although we did not find a significant correlation between survey responses and time between when the stay-at-home order was issued and survey completion, it is still possible that responses to the lifestyle change questions may have differed depending on when they were given the survey, as restrictions may have eased and respondents may be more accustomed to the “new normal”. In addition, our study was focused on consumption of combustible cigarettes and it is possible that patterns of e-cigarette/vaping use may have increased during the pandemic [14,31]. Moreover, there is evidence of increased coping-related alcohol consumption early in the COVID-19 pandemic [32]. Thus, future work should evaluate patterns of use of multiple tobacco products as well as other substances. Although the current sample size was not large enough to examine the relationship between successful cessation and demographic and psychological factors such as income, social support, anxiety, and depression symptoms, this would be an important focus of future research. In addition, the survey was administered differently across the two sites (i.e., via phone interview at the Penn site and via online at the UB site). Although it is possible that methodological differences could have influenced responses, there were no substantial differences in the length and quality of responses across sites. Lastly, our survey was cross-sectional, and a longitudinal design would have allowed for testing causal relationships between stay-at-home orders and the ability to quit smoking and a more precise assessment of changes in self-efficacy from baseline.

## 5. Conclusions

Although this study was exploratory, these data can be used to generate future hypotheses regarding how COVID-19 and restrictions to socially distance and stay home affect individuals’ mental health and substance use behaviors. Our data suggest that among smokers currently enrolled in a smoking cessation program, the stay-at-home orders enacted during COVID-19 may have altered their perception of self-efficacy for quitting smoking. Specifically, some found that the pandemic-related restrictions made it easier to quit smoking while others found it more difficult. Implications of these findings can guide future research to identify individuals who may be at risk for decreased motivation and may benefit from interventions to increase motivation. These findings suggest that changes in motivation to quit during the pandemic were largely related to perceptions of behavioral beliefs about smoking and self-efficacy. Given the recent surge in cases and the uncertainty around whether new stay-at-home orders will be issued and/or lifted, and when a vaccine will be widely available, these data may help identify individuals at greater risk for relapse and suggest targets for intervention.

## Figures and Tables

**Table 1:** Demographics and smoking characteristics across both sites (*n* = 42).

	Penn (*n* = 17)	Buffalo (*n* = 25 ^a^)	Total (*n* = 42)	*p*-Value
Sex (n, % female)	11, 65%	12, 48%	23, 55%	0.29
Race (n, %)				<0.001
Black/African-American	12, 71%	1, 4%	13, 32%	
White	4, 24%	23, 96%	27, 66%	
More than one race	1, 6%	0	1, 2%	
Age	49.9, 9.9	53.2, 8.7	51.8, 9.3	0.27
Income (n, %)				0.002
<USD 35,000	12, 71%	6, 24%	18, 43%	
USD 35,000–49,999	4, 24%	3, 12%	7, 17%	
USD 50,000–74,000	0	3, 12%	3, 7%	
>USD 75,000	1, 6%	13, 52%	14, 33%	
Education (n, %)				0.35
Some high school	3, 18%	1, 4%	4, 10%	
High school grad/GED	5, 29%	5, 20%	10, 24%	
Some college or technical school	5, 29%	12, 48%	17, 40%	
College grad or beyond	4, 24%	7, 28%	11, 26%	
Marital status (n, %)				0.006
Never married	6, 35%	0	6, 14%	
Married	2, 12%	13, 52%	15, 36%	
Divorced/Separated	5, 29%	8, 32%	13, 31%	
Widowed	1, 6%	2, 8%	3, 7%	
Marriage-like relationship	3, 18%	2, 8%	5, 12%	
Employment status (n, %)				0.042
Full-time	4, 24%	14, 58%	18, 44%	
Part-time	4, 24%	1, 4%	5, 12%	
Retired/Not currently employed	9, 53%	9, 38%	18, 44%	
Smoking Rate (cigarettes per day)	14.1, 5.6	20.7, 6.05	18, 6.75	<0.001
Nicotine Dependence Score (FTND)	5.1, 1.9	5.9, 1.9	5.6, 1.95	0.19
Age started smoking	17, 4.5	18.2, 3.8	17.7, 4.2	0.35
No. quit attempts >24 H	2.5, 3.0%	3.7, 3.3%	3.3, 3.3	0.30
Duration of longest quit attempt (n, %)				0.19
Less than a week	7, 41%	3, 12%	10, 24%	
1 week to 1 month	2, 12%	4, 16%	6, 14%	
1 month to 1 year	4, 24%	8, 32%	12, 29%	
A year or more	4, 24%	10, 40%	14, 33%	
PHQ-2 Score (range 0–5)	1.2, 1.5	0.74, 1.0	0.95, 1.2	0.21
GAD-2 Score (range 0–6)	1.3, 1.9	1.1, 1.5	1.2, 1.7	0.70

Note. Values are mean, standard deviation unless otherwise noted. ^a^ Two subjects only partially completed the survey. All available data were included. USD = US dollar. GED = General Educational Development. PHQ-2 = Patient Health Questionnaire 2-item scale. GAD-2 = Generalized Anxiety Disorder 2-item scale.

**Table 2:** Impact of COVID-19 variables.

	Not at All	Slightly	Moderately	Very	Extremely
Worried about getting COVID-19?	10, 24%	11, 26%	11, 26%	8, 19%	2, 5%
Worried about friends/family getting COVID-19?	6, 14%	13, 31%	6, 14%	9, 21%	8, 19%
Worried about your physical health because of COVID-19?	17, 40%	8, 19%	9, 21%	5, 12%	3, 7%
Worried about your mental health because of COVID-19?	13, 31%	10, 24%	13, 31%	5, 12%	1, 2%
How stressful are restrictions on leaving home?	8, 19%	13, 31%	14, 33%	4, 10%	3, 7%
Financial problems due to COVID-19?	17, 40%	10, 24%	7, 17%	5, 12%	3, 7%
Concerned about the stability of your living situation?	24, 57%	9, 21%	7, 17%	1, 2%	1, 2%
	**Rarely**	**Occasionally**	**Often**	**Most of the Time**	
How often do you read/talk about COVID-19?	2, 5%	14, 33%	14, 33%	12, 29%	
	**1–2 Days/Week**	**3–4 Days/Week**	**5–6 Days/Week**	**Every Day**	
Time spent outside of home	21, 50%	11, 26%	5, 12%	5, 12%	
	**A Lot Less**	**A Little Less**	**About the Same**	**A Little More**	**A Lot More**
Amount of contact with people outside home (vs. before COVID-19)	36, 86%	4, 10%	1, 2%	1, 2%	0
	**No Difficulty**	**Little Difficulty**	**Moderate Difficulty**	**A Lot of Difficulty**	
Difficulty following social distancing guidelines	23, 55%	15, 36%	3, 7%	1, 2%	
	**A Lot Worse**	**A Little Worse**	**It’s the Same**	**A Little Better**	**A Lot Better**
Has quality of relationships with family changed?	0	3, 7%	32, 76%	5, 12%	2, 5%
Has quality of relationships with friends changed?	2, 5%	7, 17%	31, 74%	2, 5%	0

Note. All values are n, %. SHO = stay-at-home order. There were no significant site differences for any variable listed above. All items taken from Coronavirus Health Impact Survey (CRISIS).

**Table 3:** Impact of COVID on smoking.

	Not at All	Somewhat	Quite a Bit	Extremely
COVID-19 impacted motivation to quit ^1^	19, 46%	9, 22%	9, 22%	4, 10%
COVID-19 impacted ability to quit ^1^	21, 51%	12, 29%	5, 12%	3, 7%
Cigarettes helped me cope since SHO ^2^	1, 10%	3, 30%	3, 30%	3, 30%
COVID-19 has affected my ability to obtain cigarettes ^3^	14, 64%	2, 9%	2, 9%	0
COVID-19 has affected social support during my quit attempt ^4^	29, 73%	9, 23%	2, 5%	0

Note. All values are n, %. There were no significant site differences for any variable listed above. SHO = stay-at-home order. ^1^ *n* = 41 due to missing data point; ^2^ *n* = 10 because only participants who were currently smoking were asked this question; ^3^ *n* = 22 because 18 people said that they had not tried to obtain cigarettes; ^4^ *n* = 40 due to missing data.

## Data Availability

The data presented in this study are available on request from the corresponding author.

## Supplementary Materials

The following are available online at https://www.mdpi.com/1660-4601/18/2/505/s1. Questions to subjects.
