# Supplementary material for: The Perceived Impact of COVID-19 among Treatment-Seeking Smokers: A Mixed Methods Approach
Source: Int J Environ Res Public Health. 2021 Jan 9;18(2):505. doi: 10.3390/ijerph18020505 (PMC7826630; doi:10.3390/ijerph18020505)
Supplement: Supplementary file 1 [file ijerph-18-00505-s001.pdf]

Instructions: Questions to subjects are in **bold** and should be read aloud along with response options. Word in *italics* are prompts for the interviewer and should not be read aloud.

COVID-19 IMPACTS ON SMOKING

1. **What was your smoking status the week leading up to March 16th, before the state's Stay at Home order was issued?**
    a. Not smoking at all
    b. Smoking some days
    c. Smoking everyday

    If a → skip to #2
    If b → go to 1a
    If c → skip to 1b

    1a. How many days per week were you smoking? _______

    1b. On days you were smoking, how many cigarettes per day did you smoke? ______

2. **What is your current smoking status?**
    a. Not smoking at all
    b. Smoking some days
    c. Smoking everyday

    If a → skip to #3
    If b → go to 2a
    If c → skip to 2b

    2a. How many days per week do you smoke? _______

    2b. On days you smoke, how many cigarettes per day did you smoke? _______

3. **Has the Coronavirus/COVID-19 crisis impacted your <u>motivations</u> for quitting smoking/staying quit?**
    a. Not at all
    b. Somewhat/ A little
    c. Quite a bit
    d. Extremely

    If a → Skip to #4
    If b-d → Go to 3a

    3a. How has your motivation changed (increased/decreased) or has it stayed the same?
    Probe: Why do you think your motivation has increased/decreased/stayed the same?

4. **Has the Coronavirus/COVID-19 crisis in your area impacted your <u>ability</u> to quit smoking/stay quit?**
    a. Not at all
    b. Somewhat/ A little
    c. Quite a bit
    d. Extremely

If a → Skip to #5
If b-d → Go to 4a

4a. Is it more or less difficult to quit with the Stay at Home order?
    a. More
    b. Less
    c. Both

In what ways is it more/less difficult?

For the following questions, please rate the degree to which the statement is true for you:

***If participant is NOT currently smoking*, skip to #7.**
***If participant is currently smoking*, go to #5.**

5. **Cigarettes have helped me stay calm (cope) since the Coronavirus/COVID-19 crisis in my area**.
    a. Not at all
    b. Somewhat/ A little
    c. Quite a bit
    d. Extremely

If a → Skip to #6
If b-d → Go to 5a

5a. Can you tell me a little more about how cigarettes have helped you cope/stay calm?

6. **The Coronavirus/COVID-19 crisis in my area has affected my ability to obtain cigarettes.**
    a. Not at all
    b. Somewhat/ A little
    c. Quite a bit
    d. Extremely

If a → Skip to #7
If b-d → Go to 6a

6a. Are they more or less available?
    a. More
    b. Less
    c. No change

7. **The Coronavirus/COVID-19 crisis in my area has affected my social support during my quit attempt?**
    a. Not at all
    b. Somewhat/ A little
    c. Quite a bit
    d. Extremely

If a → Skip to #8
If b-d → Go to 7a

7a. In what ways has your social support been affected?
Probe: How have your interactions with other people impacted your smoking?

8. **How have your smoking triggers/high risk situations changed since the start of the Coronavirus/COVID-19 crisis in your area**?

    Probe: In what way have they changed?
    Probe: Have any new triggers emerged? Yes/No
        If yes: What new triggers have emerged?

9. **Since the Stay at Home order was issued in what ways have you had to change or adjust your quit smoking strategies?**

    Probe: What quit smoking strategies are you currently using? Which of these were you using before the Coronavirus/COVID-19 crisis in your area?

10. **Of the people you live with or interact with on a daily basis, how many of them are current smokers? _____**

    10a. How is this the same/different from before the Stay at Home order was issued on March 16th?

    I am living/interacting with:
    a. … more smokers than before.
    b. … fewer smokers than before.
    c. …the same number of smokers as before.

    10b. How has this impacted your smoking compared to before the Stay at Home order was issued?
    a. I am smoking more than before.
    b. I am smoking less than before.
    c. I am smoking the same amount/still not smoking.

11. **Since the Stay at Home order was issued, we have been doing all of our study visits and counseling via phone/remotely. How has this experience been for you?**
